# Binding time: Investigations on the integration of visual stimulus duration

**DOI:** 10.1177/17470218221140751

**Published:** 2022-12-21

**Authors:** Katrin Köllnberger, Johanna Bogon, Gesine Dreisbach

**Affiliations:** 1 Department of Psychology, University of Regensburg, Regensburg, Germany; 2 Media Informatics Group, University of Regensburg, Regensburg, Germany

**Keywords:** Feature binding, partial repetition costs, temporal binding, timing, time perception

## Abstract

The perception of and reaction to objects creates bindings of (object) features and responses, also called event files. In this context, *time* is a so far understudied feature. We conducted four experiments to investigate whether the duration of visual stimuli is also integrated into such event files. Experiments 1, 2, and 4 used a simple colour classification task and in Experiment 3 the location of a stimulus had to be classified. In all Experiments, the presentation duration of the stimuli (coloured circles) was either short (20 ms) or long (300 ms). We expected partial repetition costs as an indicator of binding. That is, performance should be better when both colour (Experiment 3: location) and duration repeat or alternate relative to partial repetitions. Results showed no partial repetition costs in Experiments 1 and 3, indicating no integration of duration into visual event files. Experiments 2 and 4 revealed partial repetition costs. Performance was better when Colour and Duration repeated compared with a partial repetition. What distinguishes the latter two experiments from the former is that the coloured stimuli could change their presentation location. The results of all four experiments show a pattern that duration can be integrated into visual event files depending on two criteria: The experimental context holds the possibility of a location change of the target stimulus (Experiments 2 and 4) and the location itself is not response relevant (Experiment 3). The role of location changes for the integration of temporal stimulus features into visual event files is discussed.

## Binding time: Investigations on the integration of visual stimulus duration

The human brain processes different features of an event (like the colour of a cup, its location, and its associated actions) in a widely distributed fashion (Felleman & van Essen, 1991; Jeannerod, 1999; Mesulam, 1998; van Essen et al., 1992). And still (at least most of the times) we are able to reach for the correct cup to take a sip and avoid accidentally drinking from a stranger’s cup instead. The coherent perception together with the appropriate action is achieved by temporary bindings of stimulus and response features, so-called event files (Hommel, 1998). Feature binding is a ubiquitous phenomenon and has been found for different modalities and features like colour, sound, shape, location, to only name a few (e.g., Delogu et al., 2014; Hommel & Colzato, 2009; Maybery et al., 2009; see Frings et al., 2020, for a review). Although time is a pervasive feature in a dynamically changing environment and is often part of visual and auditory events, the integration of temporal features has hardly been studied. Bogon, Thomaschke, and Dreisbach (2017) brought up first evidence for the integration of stimulus duration into auditory event files. Because duration is a defining feature of auditory events—having a natural beginning and end—the question arises whether duration is also bound to visual events that, by nature, do not necessarily have a definite duration (e.g., objects in the physical world). Therefore, in this study, we aim to investigate whether and under what conditions stimulus duration is also integrated into visual event files.

### Object / event files and partial repetition costs

According to Treisman’s *feature integration theory* (Kahneman et al., 1992; Treisman, 1988, 1996; Treisman & Gelade, 1980), features belonging to the same object are bound together in an object file, which contains the traces to the distributed perceptual feature representations. In most cases, such binding processes are not only restricted to the integration of perceptual stimulus features, but also to response-related features. Therefore, the concept of an object file was extended to an event file, which additionally contains action-related features (Hommel, 1998, 2004). Once built, the event file persists for a short period of time. With our environment being composed of countless consecutive events with varying perceptual and action-related features, these temporary feature compounds can lead to benefits or interference during action control. Consequently, binding is typically investigated with experiments assessing the effect of a previously created and persisting event file on a subsequent event (e.g., Bogon, Eisenbarth, et al., 2017; Frings et al., 2007; Hommel, 1998; Kleinsorge, 1999; Mayr & Buchner, 2006; Rothermund et al., 2005). Take the following example: Participants are asked to react to the colour (red or green) of a shape (circle or square) with a left or right response. If binding takes place, partial repetition costs can be measured in consecutive trials whenever one feature (colour or shape) is repeated and the other is not (e.g., a red circle is followed by a red square), whereas no such costs occur when either both features repeat (red circle followed by another red circle) or both change (red circle is followed by a green square). The reasoning goes as follows: The repeated feature causes a retrieval of the still persisting event file from the previous trial which interferes with the correct response of the current trial, leading to higher RTs or error rates. A complete repetition, in contrast, leads to a benefit or at least no costs in performance because the previously formed event file matches completely with the current one. A complete alternation behaves in a comparable way because no interference with the previously formed representation occurs. This behavioural pattern (statistically an interaction of Feature A [e.g., shape] × Feature B [e.g., colour]) is known as *partial repetition costs* and is interpreted as evidence for feature integration (Frings et al., 2013; Hommel, 1998, 2004; Kahneman et al., 1992; Mayr et al., 2011; van Dam & Hommel, 2010; Zmigrod & Hommel, 2009, 2010).

For the visual modality, binding has already been shown for features such as shape, colour and orientation (Hommel, 1998; Kahneman et al., 1992; van Dam & Hommel, 2010), picture identity (Frings et al., 2013), letter and word identity (e.g., Kahneman et al., 1992; Rothermund et al., 2005; Tipper & Cranston, 1985), and face features (Fitousi, 2017). There is also evidence for the integration of auditory stimulus features into event files, for example, for features such as pitch and volume (Zmigrod & Hommel, 2009, 2010), sound identity (Maybery et al., 2009; Mayr et al., 2011), and voice-related features like gender and affect (Bogon, Eisenbarth, et al., 2017). Furthermore, binding also occurs for combinations of features from different sensory modalities (e.g., Zmigrod et al., 2009; see Spence & Frings, 2020, for a review). Moreover, binding effects—though partly weaker—have also been shown for task-irrelevant features (e.g., Fitousi, 2017; Hommel, 1998; Hommel & Colzato, 2004; Moeller et al., 2012; Moeller, Frings, & Pfister, 2016; Zmigrod & Hommel, 2009).

### Integration of duration

It is striking that in this enormous number of already investigated features, the feature *duration* is still mostly uninvestigated. In contrast to most features, that are dependent on specific stimulus, action, or effect modalities, durations are ubiquitous. Whereas features like loudness and colour are almost exclusively restricted to the auditory and visual modality, durations are an integral part of every auditory, visual or tactile stimulus (e.g., the presentation duration), every action (e.g., the duration of a key press; the duration of a vocalisation), and every auditory, visual, or tactile action effect (e.g., the effect duration). Despite time being inherent and essential to any stimulus-response event, the integration of duration is by no means a trivial issue. Mechanisms that work for nontemporal features cannot easily be transferred to features involving the flow of time (cf. anisotropy of time, Riemer, 2015; Riemer et al., 2012). One fundamental difference is that temporal features are dynamic by nature, in contrast to colour, for example, which is static. Naming the colour of a presented object is possible immediately from the beginning of its presentation, whereas the duration of an object is not known until the presentation ends. That is, duration itself is constantly updated. In other words, during the ongoing event, the event duration automatically changes its specification over time. This is the challenging aspect for integrating duration into event files: Almost all nontemporal event features that are steadily present from the first occurrence of the event can be integrated from the first moment the event appears. This is not possible for the duration of the event: During the persistence of the event, the duration is constantly redefined. Therefore, strictly speaking, binding the current duration at a certain timepoint makes little sense because the bound duration immediately gets obsolete as the event persists. Moreover, the recent finding that the temporal order of integrated elements of an event is not part of the event file representation (Moeller & Frings, 2019) additionally suggests the nonintegration of temporal features into event files.

Despite all this, the integration of duration representations seems to make sense because in many cases they are mandatory for the identification, differentiation, and processing of events. In music, for example, duration plays a crucial role. A rhythm results from a pattern of tones that vary in duration or are presented in a delayed fashion. The time interval between consecutive tones is important for the perception of rhythm. If the interval is too short (less than 100 ms), the tones are perceived by the listener as only one tone. In contrast, if the interval between tones is too long (more than 1500 ms), it leads to difficulties in grouping the tones into a sonorous pattern. Moreover, the individual temporal organisation of musical elements leads to the uniqueness of a song and is thus an important factor for music recognition (Krumhansl, 2000). Furthermore, duration is an important component of speech. For example, the duration of a spoken syllable influences the processing and perception of that syllable and thus has an impact on phonetic classification. The American English spoken word “dessert” [dɪˈzɝːt] has a clearly different meaning than the, very similarly written, word “desert” [ˈdɛzɚt]. Speech duration influences the intelligibility and identification of spoken words (Miller & Volaitis, 1989).

First findings for the integration of temporal stimulus features into auditory event files were shown by Bogon, Thomaschke, and Dreisbach (2017). In two experiments, they investigated binding effects between task-irrelevant stimulus duration and pitch (Experiment 1), and task-irrelevant stimulus duration and loudness (Experiment 2). In Experiment 1, participants had to classify the pitch (low vs. high) of four sine tones by pressing a key (left or right). The tones (high: 800 Hz, low: 400 Hz) were presented either short (50 ms) or long (200 ms). A partial repetition costs pattern was obtained: A pitch repetition combined with a duration repetition resulted in better performance than when duration alternated. During a pitch change, duration had no effect on performance. The design and procedure of Experiment 2 mirrored that of Experiment 1, with the change that participants were asked to classify loud (80 dB) and soft (40 dB) tones by keypress. Again, results showed a partial repetition costs pattern: Performance was better when both loudness and duration remained the same or both alternated, compared with a partial repetition of the features. The results of both experiments showed that the stimulus duration which was irrelevant for the task was integrated into the auditory event files.

What remains unclear, however, is to what degree these findings extend to the visual modality. If it is possible to bind duration to visual features, comparable results, to the previously shown binding of duration to auditory features by Bogon, Thomaschke, and Dreisbach (2017), should emerge. Nevertheless, it should be noted that durations of auditory and visual features may differ (see Wearden & Jones, 2021, for a review). First, the duration of a shown visual stimulus can be influenced by self-paced looking time or blinking or closing the eyes entirely. Although this is also possible with auditory stimuli, by, for example, covering the ears, it is less likely in an experimental framework as well as in real life. Simply put, looking away is easier and more common than “listening away.” Furthermore, the importance of the duration of visual and auditory stimuli might differ. As outlined above, in most auditory domains, the duration is an inherent part of the sensory event and often carries important information whereas visual stimuli in the physical world rarely do so. For example, a continuous sound of the smoke detector warns to leave the apartment, whereas a short sound may indicate low battery. For visual stimuli, it is more difficult to find examples for which duration might be relevant. Nevertheless, there are visual stimuli for which duration has an informative character. The duration of light signals on the high seas, for example, is of great importance for deciphering morse code or for assigning the light pattern of a lighthouse to its respective location. Furthermore, the perceived instantaneous duration of the green phase of a traffic light has an informative character. A perceived longer duration of the green phase would rather prompt the driver to prepare for a braking reaction than a perceived shorter duration of the green phase (the traffic light has only recently turned green).

Regarding the processing of time, a superiority of the auditory modality over the visual modality has been observed (see Grondin, 2003, 2010; Wearden & Jones, 2021, for a review). As an example, auditory events are perceived longer than visual events of the same duration (Goldstone & Lhamon, 1974; Walker & Scott, 1981; Wearden et al., 1998). Nevertheless, the traffic light example exemplifies that the duration of visual events has implications on our actions and suggests its integration.

### Present study

The purpose of the present study is to investigate whether the irrelevant stimulus duration is likewise integrated into visual event files. Four experiments were conducted for this purpose. Experiment 1 used a colour classification task to investigate whether duration is bound to colour. Adapted from the study by Bogon, Thomaschke, and Dreisbach (2017), the two coloured stimuli were presented either for a short (20 ms) or for a longer (300 ms) duration. The colour feature was task-relevant, and the duration feature was task-irrelevant. If duration is integrated into visual event files, sequential analysis should reveal a partial repetition costs pattern. Repetition of duration from trial *n* − 1 to trial *n* paired with repetition of colour, should result in better performance compared with repetition of duration paired with a change in colour. Similarly, a change of both features should produce better performance than a partial repetition/change of features. The second experiment examines the role of duration on the integration of stimulus duration into visual events when embedded in a setting more relevant for everyday life. The third experiment examines the binding of duration to location. In the fourth experiment, the binding of duration to colour, as well as to location, is examined in more detail. Analogous to Bogon, Thomaschke, and Dreisbach (2017), duration was task irrelevant in all four experiments.

## Experiment 1

### Material and methods

#### Participants

A power analysis (G*Power 3.1.9.7, Faul et al., 2007) revealed that to detect a two-way interaction effect size of $\eta_{p}^{2}$ = .42 (the minimum interaction effect size from Bogon, Thomaschke, & Dreisbach, 2017; used option: effect size specification as in SPSS), with a power of 1 − β = .95 and α = .05, a minimum sample size of *N* = 21 would be necessary. To ensure that the minimum number of required participants would be obtained even after possible exclusion of participants, we aimed for a minimum number of 30 participants for the acquisition of each experiment. Consequently, all experiments exceed the minimum number of 21 participants.

The participants in all four experiments were students at the University of Regensburg and participated for course credit. They all gave written informed consent prior to the experiment in accordance with the ethical standards of the national research committee and with the 1964 Helsinki declaration and its later amendments. Furthermore, all participants reported normal or corrected-to-normal vision and had intact colour vision (relevant for Experiments 1, 2, and 4) as indicated by the electronic 10 plates version of the Ishihara test for colour blindness (Birch, 1997).

In Experiment 1, two of the 30 female participants had to be excluded due to mean RTs lying more than three interquartile ranges above the third quartile of the sample distribution (exclusion criteria were set a priori). For the remaining sample, the mean age was 22.46 (*SD* = 3.21) years with a range from 18 to 33 years and one was left-handed (self-report).

#### Apparatus and stimuli

**Figure 1.**
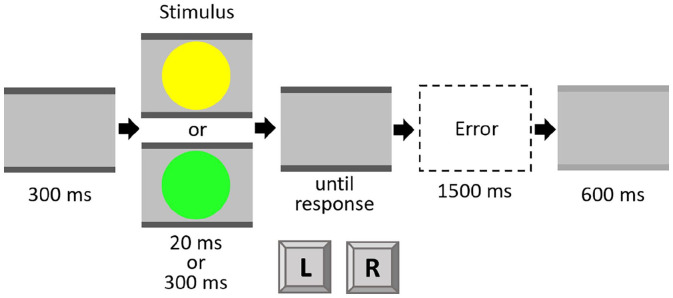
Trial procedure in Experiment 1. Only one stimulus (yellow or green) was shown in a given trial. Stimuli are not drawn to scale.

The experiment was run in E-Prime (Version 2.0, Psychology Software Tools, Sharpsburg, USA). The stimuli were made up of a combination of two coloured circles (green and yellow) of two durations (20 ms and 300 ms), resulting in a total of four stimuli with a size of 1.9° × 1.9° visual angle from a distance of approximately 60 cm on a grey background. Instructions and stimuli were shown on a standard colour monitor (19″ diagonal; 1440 × 900 px; 60 Hz). Participants had to react to the colour of the stimulus with a left and right response key (“Y” and “M” keys on a standard QWERTZ keyboard), positioned centrally in front of the participant. Assignment of colour to response was counterbalanced between participants. The stimuli were always presented at the centre of the screen with two grey bars above and below. The grey bars without a stimulus served as fixation. At the intertrial interval (ITI), the bars changed to a light grey colour (see Figure 1).

#### Design and procedure

Each trial started with fixation bars of 300 ms duration. These bars were constantly visible throughout the whole trial. Then, the coloured target circle was presented either for 20 ms or for 300 ms,^1^ followed by a blank screen that was visible until the response was given. Participants were also able to give a response during the stimulus presentation. In this case, the blank screen was skipped. After an ITI of 600 ms, the next trial started. When participants responded using the wrong button, an error message appeared for 1500 ms (see Figure 1). The experiment consisted of one practice block of 20 trials and three experimental blocks of 80 trials each. The order of trials was randomised with the constraint that in the experimental blocks each factor combination (Colour Sequence × Duration Sequence) appeared at least 18 times. Participants were instructed to respond as fast and accurately as possible.

A 2 × 2 design was used, with the within-subject factors Colour (repetition vs. shift) and Duration (repetition vs. shift). If duration is bound to the stimulus colour, we should find a Colour × Duration interaction.

Raw data files associated with this article can be found online (https://doi.org/10.5283/epub.53290).

### Results and discussion

We analysed data from the three experimental blocks. The first trial of each block was excluded from analysis. Moreover, error trials (3.95%), trials following an error trial (4.05%), trials with extreme RTs < 100 ms and > 8000 ms (0.01%), and trials with RTs deviating more than 3 *SDs* from the individual condition mean (1.19%) were excluded from the reaction time (RT) analysis (Bush et al., 1993).

In all experiments, data were analysed using SPSS statistical software (IBM Corp., 2017). All Bayes factors were determined using the MorePower (Campbell & Thompson, 2012) programme. The categorization of Bayes factors is based on the work of Wagenmakers et al. (2011) and Wetzels et al. (2011).^2^ The results were visualised using R (R Core Team, 2022; R version 4.2.1) using the *ggplot2* package (Version 3.3.6, Wickham, 2022).

**Figure 2.**
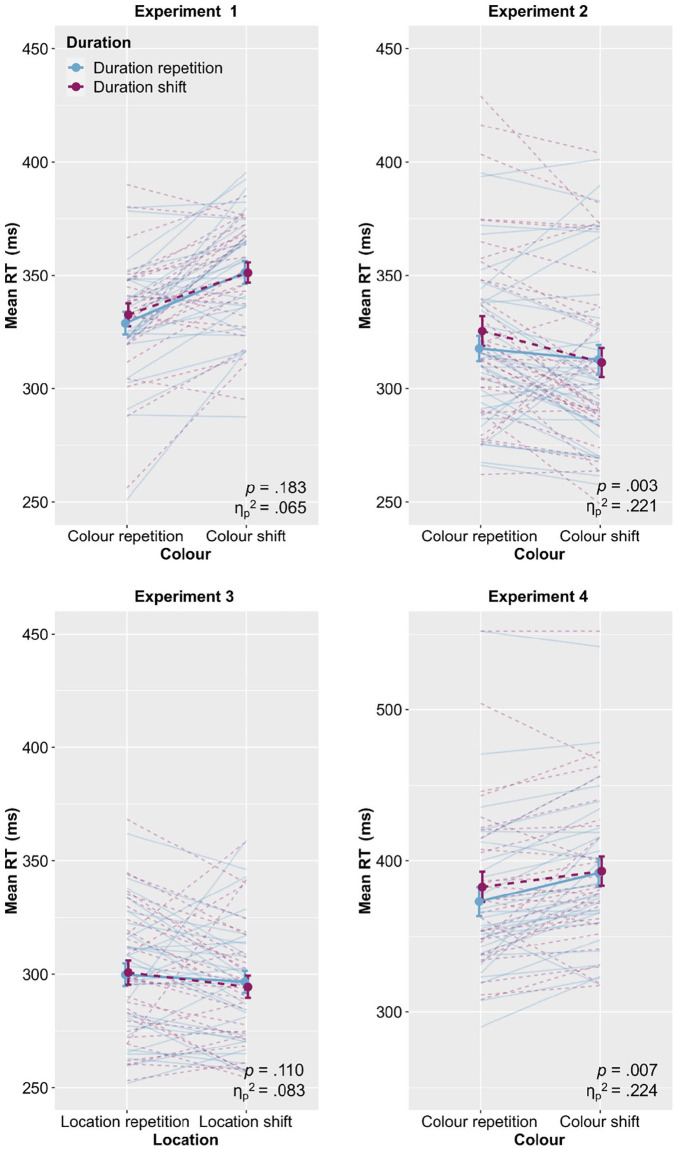
Mean RTs as a function of the respective factors of Experiments 1 to 4. Upper left panel displays the Colour × Duration interaction from Experiment 1 and the upper right panel shows the Colour × Duration interaction from Experiment 2. Lower left panel displays the Location × Duration interaction from Experiment 3 and the lower right panel illustrates the Colour × Duration interaction from Experiment 4. The solid lines represent duration repetitions and the dashed lines represent duration shifts. The individual slope is shown for each participant and error bars provide standard errors. Experiments 1–3 are plotted with the same scale and Experiment 4 uses a different scale to make all slopes visible. The *p* values and effect size estimates refer to the Feature × Duration interaction. RT: reaction time.

Figure 2 (upper left panel) plots mean RTs as a function of Colour and Duration. We conducted a 2 (Colour: repetition vs. shift) × 2 (Duration: repetition vs. shift) analysis of variance (ANOVA) with repeated measures on both factors. This revealed a significant main effect of Colour, *F*(1, 27) = 27.8, *p* < .001, $\eta_{p}^{2}$ = .508, indicating faster responses when the colour was repeated compared with when the colour changed (331 ms vs. 351 ms). The factor Duration was not significant (*F* = 2.26, *p* = .145, $\eta_{p}^{2}$ = .077). Contrary to our hypothesis, there was no significant interaction Colour × Duration (*F* = 1.87, *p* = .183, $\eta_{p}^{2}$ = .065). The Bayes factor (BF_01_ = 2.08) of this interaction indicates anecdotal evidence for the null hypothesis.

An analogous ANOVA for errors yielded a significant main effect of Duration, *F*(1, 27) = 4.49, *p* = .044, $\eta_{p}^{2}$ = .143, indicating that, overall, duration changes were more error prone than duration repetitions (4.83% vs. 3.91%). None of the other effects were significant (all *F*s < 3.31 and *p*s > .080).

The results of Experiment 1 did not show the expected Colour × Duration interaction, neither in RT nor in error data. The absence of partial repetition costs indicates no evidence for binding of colour and duration, thus no integration of duration into visual event files. One possible reason for this lack of integration is that the duration of simple coloured circles in green and yellow in our experiment was not informative or task-relevant. That is, there was no need to process the duration, because it carried no informative value. However, as explicated above, there exist situations in which the duration of a visual stimulus is informative, as it is the case in the example of a traffic light from the introduction: The longer I see the green light, the more likely it becomes that it will turn to red, making the duration of the colour a relevant stimulus feature. To investigate whether the duration of a visual stimulus would be integrated into an event file if the duration is framed as potentially relevant, we conducted Experiment 2. More precisely, we compared binding effects between colour and duration depending on whether they were presented as part of a traffic light or not. Although the duration feature remains task-irrelevant in the traffic light version of the task, the intended association with the usually relevant signal duration of a traffic light might enhance the potential relevance of visual stimulus duration. We expected the integration of duration into visual event files in the traffic light condition but no integration in the standard condition (comparable with Experiment 1).

## Experiment 2

### Material and methods

#### Participants

One of the 37 participants was excluded, due to technical problems. For the remaining sample, the mean age was 21.3 (*SD* = 1.84) years with a range from 19 to 27 years, with 25 females and three left-handed (self-report).

#### Stimuli and procedure

**Figure 3.**
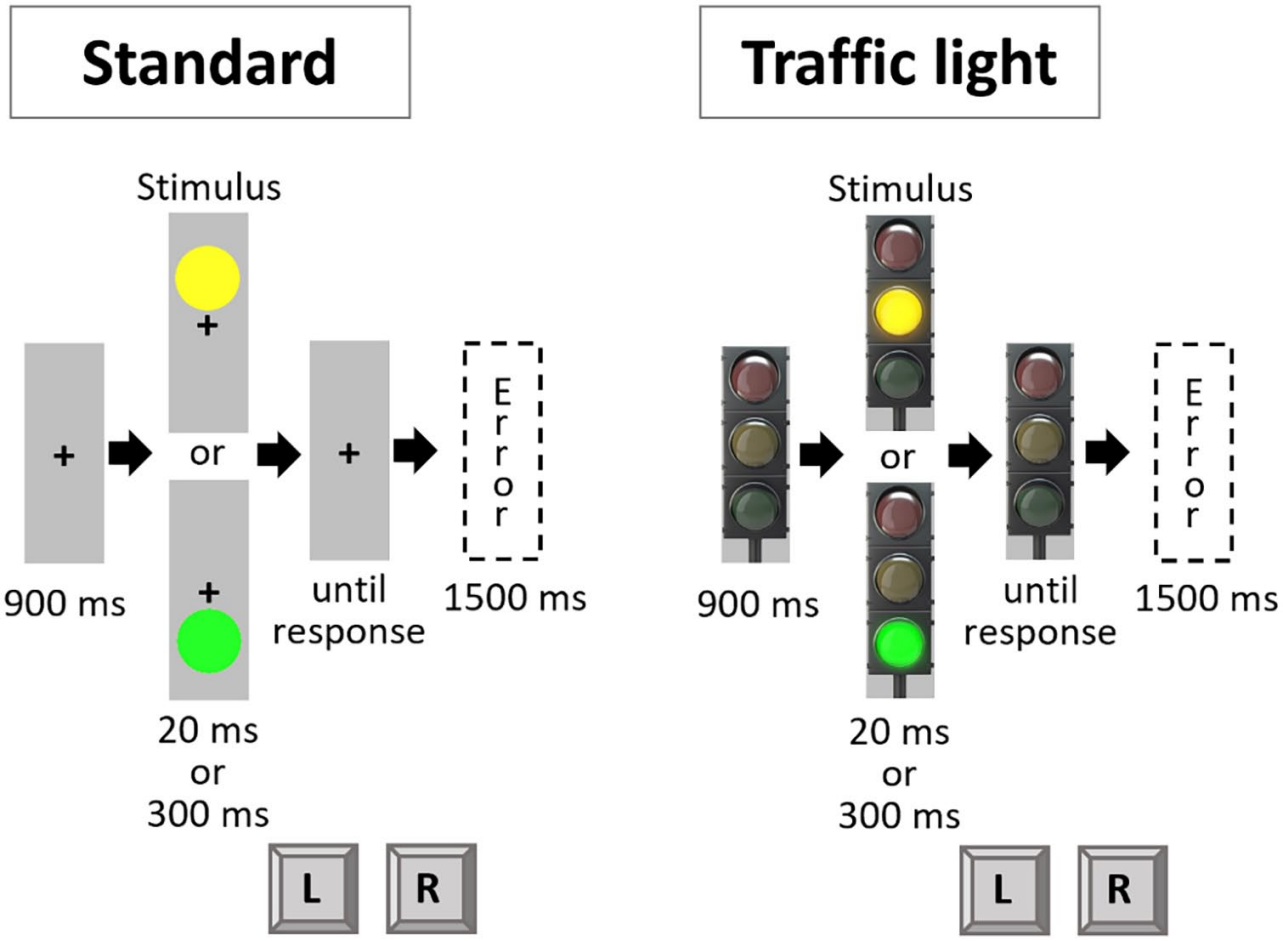
Trial procedure in Experiment 2. Left panel displays the stimuli of the standard condition, right panel displays the stimuli of the traffic light condition. Note that in the standard condition, the coloured stimuli were presented without any frame above versus below the fixation cross. Stimuli are not drawn to scale.

The procedure of Experiment 2 mirrored that of Experiment 1, except for the following modifications: Instructions and stimuli were shown on a standard colour monitor (17.6″ diagonal; 1024 × 768 px; 84 Hz). The experiment now included two kinds of stimulus framing. In one framing condition of the experiment (standard condition), the yellow circle always appeared above a newly introduced fixation cross (black, arial pt. 18, bold, always visible over the entire trial) and the green circle always appeared below. In the other framing condition of the experiment (traffic light condition), these circles were integrated into a traffic light. Thus, the size and location of the circles were the same between conditions, but the framing was different. Furthermore, an off-state traffic light served as fixation in the traffic light condition, which was again presented for the entire duration of a given trial (see Figure 3). The colours again were presented either short (20 ms) or long (300 ms).^3^ In total, the experiment consisted of two practice blocks with 20 trials each and six experimental blocks with 80 trials each. Therefore, each framing condition consisted of one practice block and three experimental blocks. The participants thus started with one practice block and three experimental blocks in one framing condition and then worked through one practice block and three experimental blocks in the other framing condition. The order of the framing condition (standard or traffic light) and the task mapping (colour to response key) was counterbalanced across participants.

A 2 × 2 × 2 design was used, with the within-subject factors Framing (standard vs. traffic light), Colour (repetition vs. shift), and Duration (repetition vs. shift). We expected a three-way interaction between all these factors. More precisely, we expected a Colour × Duration interaction in the traffic light condition but not in the standard condition.

### Results and discussion

Preprocessing was exactly the same as in Experiment 1. Error trials (3.21%), trials following an error trial (3.35%), trials with extreme RTs < 100 ms and > 8000 ms (0.08%), and trials with RTs deviating more than 3 *SDs* from the individual condition mean (1.27%) were excluded from the RT analysis (Bush et al., 1993).

Figure 2 (upper right panel) plots mean RTs as a function of Colour and Duration (see Table 1 for full descriptive statistics). We conducted a 2 (Framing: standard vs. traffic light) × 2 (Colour: repetition vs. shift) × 2 (Duration: repetition vs. shift) ANOVA with repeated measures on all factors. This revealed a significant main effect of Colour, *F*(1, 35) = 7.41, *p* = .010, $\eta_{p}^{2}$ = .175, indicating faster responses when the colour changed compared with when the colour was repeated (312 ms vs. 322 ms). The main effect of Duration also reached significance, *F*(1, 35) = 10.5, *p* = .003, $\eta_{p}^{2}$ = .230, indicating faster responses when the duration was repeated compared with when the duration changed (315 ms vs. 318 ms). The factor Framing was not significant (*F* = 1.55, *p* = .222, $\eta_{p}^{2}$ = .042).

**Table 1.** Mean response times in ms, as a function of Frame, Colour, and Duration for Experiment 2.

	Standard		Traffic light	
	Colour repetition	Colour shift	Colour repetition	Colour shift
RT				
Duration repetition	319 (5.69)	318 (6.76)	316 (6.58)	307 (6.89)
Duration shift	325 (7.37)	317 (6.87)	325 (7.27)	306 (6.66)

The respective standard errors are in brackets. RT: reaction time.

In contrast to Experiment 1, here the analyses revealed a significant interaction Colour × Duration, *F*(1, 34) = 9.92, *p* = .003, $\eta_{p}^{2}$ = .221, indicating partial repetition costs: Responses were faster when colour and duration were repeated compared with when colour repeated and duration changed (318 ms vs. 325 ms). The Bayes factor (BF_10_ = 14.9) of this interaction indicates strong evidence for H_1_. However, and in contrast to our hypothesis, this effect was not further modulated by Framing. The corresponding three-way interaction Framing × Colour × Duration was far from significant (*F* = 0.379, *p* = .542, $\eta_{p}^{2}$ = .011).^4^ The Bayes factor (BF_01_ = 4.94) of this interaction suggests substantial evidence for H_0_.

Moreover, there was a significant Framing × Colour interaction, *F*(1, 35) = 7.61, *p* = .009, $\eta_{p}^{2}$ = .179, indicating faster responses for colour changes in the traffic light condition compared with colour changes in the standard condition (307 ms vs. 317 ms). The interaction Framing × Duration was not significant (*F* = 0.586, *p* = .449, $\eta_{p}^{2}$ = .016).

To look into potential order effects of the framing condition, we conducted a four-factorial ANOVA with the additional factor Order (traffic light first vs. standard first). The interaction Framing × Colour × Duration × Order was not significant (*F* = 1.75, *p* = .195, $\eta_{p}^{2}$ = .049).

None of the effects in the analogous ANOVA for errors was significant (all *F*s < 1.43 and *p*s > .240). As in Experiment 1, the mean error rate (3.43%) was low.

The results of Experiment 2 did not show the expected three-way interaction Framing × Colour × Duration, neither in RT nor in error data. Instead, the RT results indicate the integration of duration into visual event files independently of the framing condition. The traffic light task as well as the standard task revealed partial repetition costs: Better performance when colour and duration repeated compared with a colour repetition combined with a duration change.

The control analysis with the fourth factor Order eliminated the argument that the character of the daily life relevance was transferred from the traffic light condition to the standard condition, for participants who had to perform the traffic light condition first. Despite this, one could argue that the used colours themselves evoke an association with a traffic light, so that the standard condition was already perceived as a traffic light. However, the results of Experiment 1, where we used the same colours but did not find an interaction with the duration, speak against this argument. All the more the question arises why duration is bound to colour in Experiment 2 but not in Experiment 1. One critical difference between Experiment 1 and the standard condition in Experiment 2 might have been the location of the stimuli. Experiment 1 used centred circles, whereas in Experiment 2 circles were presented above or below the fixation. Moreover, the yellow circle was always presented above and the green circle always below fixation. This assignment was due to the structure of a traffic light. As a consequence, each colour change was always also a location change. This leaves two potential explanations for the contradictory results between Experiment 1 and the standard condition in Experiment 2: Either, the location change that always coincided with the colour change made duration potentially meaningful, and thus strengthened the binding between duration and colour. Or, it was not the colour that was bound to duration in the first place but the location. If the latter is true, we should find the same binding effect between location and duration while keeping the stimulus colour constant. In Experiment 3, we therefore manipulated the location of the stimulus and its duration but not the stimulus colour.

## Experiment 3

### Material and methods

#### Participants

Seven of the 38 participants had to be excluded. Four due to technical problems, two due to problems with the screen brightness, and one due to strong fatigue. For the remaining sample, the mean age was 21.4 (*SD* = 2.53) years with a range from 18 to 28 years, with 21 females and one left-handed (self-report).

#### Stimuli and procedure

**Figure 4.**
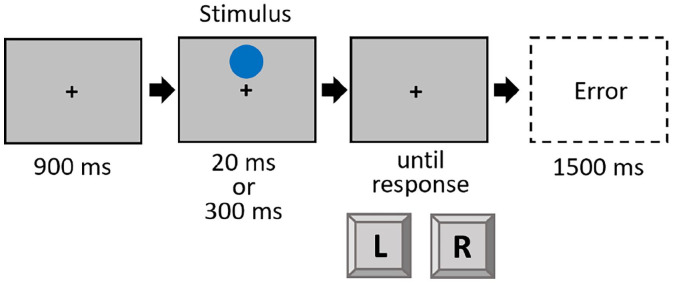
Trial procedure in Experiment 3. This is an example of a trial in which the stimulus is presented above the fixation cross and requires the correct response “up.” In all trials, the stimulus (blue circle) could appear either above or below the fixation cross. Stimuli are not drawn to scale.

The procedure of Experiment 3 mirrored that of Experiment 1, with the following exceptions: Instructions and stimuli were shown on a standard colour monitor (17.6″ diagonal; 1024 × 768 px; 84 Hz). The stimulus was a blue circle, which could appear either above or below a newly introduced fixation cross (black, arial pt. 18, bold, always visible over the whole trial). Analogous to the preceding experiments, the stimuli could appear either short (20 ms) or long (300 ms)^5^ (see Figure 4). Participants worked through a practice block of 20 trials and three experimental blocks of 80 trials each. Participants reacted to the location of the stimulus (up vs. down) with a left or right response. The response mapping (location to response key) was counterbalanced across participants.

A 2 × 2 design was used, with the within-subject factors Location (repetition vs. shift) and Duration (repetition vs. shift).

### Results and discussion

Preprocessing was the same as in the preceding experiments. Error trials (3.04%), trials following an error trial (3.31%), trials with extreme RTs < 100 ms and > 8000 ms (0.03%), and trials with RTs deviating more than 3 *SDs* from the individual condition mean (1.30%) were excluded from the RT analysis (Bush et al., 1993).

Figure 2 (lower left panel) plots mean RTs as a function of Location and Duration. We conducted a 2 (Location: repetition vs. shift) × 2 (Duration: repetition vs. shift) ANOVA with repeated measures on both factors. This revealed neither any main effects (both *p*s > .247) nor a Location × Duration interaction, *F*(1, 30) = 2.72, *p* = .110, $\eta_{p}^{2}$ = .083. The Bayes factor (BF_01_ = 1.45) suggests anecdotal evidence for H_0_.

An analogous ANOVA for errors yielded a significant main effect of Location, *F*(1, 30) = 5.22, *p* = .030, $\eta_{p}^{2}$ = .148, indicating that, overall, location changes were more error prone than location repetitions (3.73% vs. 2.71%). None of the other effects was significant (all *F*s < 0.518 and *p*s > .477). As in the experiments before, the mean error rate (3.21%) was low.

The absence of a Location × Duration interaction in Experiment 3, for either RTs or error rates, rules out the latter, of the two previously mentioned explanations. The results did not indicate binding between location and duration. Therefore, the interaction between colour and duration in Experiment 2 cannot be explained by the fact that, due to the experimental setup, colour was equated with location and possibly a binding to location instead of colour took place. Nevertheless, the results so far still cannot explain why colour was not bound to duration in Experiment 1 but was in Experiment 2. As mentioned before, there is another possible explanation: in Experiment 2 a change of colour was always a change of location at the same time. This location change might have made the duration potentially meaningful, and thus strengthened the integration of colour and duration in one event file. To clarify this, we conducted Experiment 4, with an experimental setting that allows colour and location to be considered independently of each other. If the change of location is responsible for the binding between colour and duration, we should find binding effects between colour and duration (as in Experiment 2) and none for location and duration (as in Experiment 3).

## Experiment 4

### Material and methods

#### Participants

Thirty participants took part in this experiment. The mean age was 23.0 (*SD* = 2.60) years with a range from 20 to 29 years, with 24 females and one left-handed (self-report).

#### Stimuli and procedure

**Figure 5.**
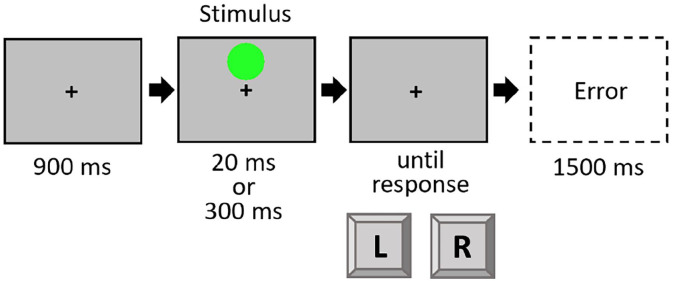
Trial procedure in Experiment 4. This is an example of a trial in which the stimulus (green circle) is presented above the fixation cross and requires a response with the response key associated with the colour green. In all trials, the stimulus (green or yellow circle) could appear either above or below the fixation cross. Stimuli are not drawn to scale.

The procedure of Experiment 4 mirrored the standard condition of Experiment 2, with the following exceptions: Instructions and stimuli were shown on a standard colour monitor (17.8″ diagonal; 1024 × 768 px; 84 Hz). The yellow and green circles could appear above as well below the fixation cross (black, arial pt. 18, bold, always visible over the whole trial). Analogous to the preceding experiments, the stimuli could appear either short (20 ms) or long (300 ms)^6^ (see Figure 5). Participants worked through a practice block of 20 trials and six experimental blocks of 80 trials each. The task mapping (colour to response key) was counterbalanced over all participants.

A 2 × 2 × 2 design was used, with the within-subject factors Colour (repetition vs. shift), Location (repetition vs. shift), and Duration (repetition vs. shift). We expected a Colour × Duration interaction, thus partial repetition costs, and no interaction of Location × Duration.

### Results and discussion

Preprocessing was exactly the same as in the preceding experiments. Error trials (4.63%), trials following an error trial (4.94%), trials with extreme RTs < 100 ms and > 8000 ms (0.01%), and trials with RTs deviating more than 3 *SDs* from the individual condition mean (1.45%) were excluded from the RT analysis (Bush et al., 1993).

Figure 2 (lower right panel) plots mean RTs as a function of Colour and Duration (see Table 2 for full descriptive statistics). We conducted a 2 (Colour: repetition vs. shift) × 2 (Location: repetition vs. shift) × 2 (Duration: repetition vs. shift) ANOVA with repeated measures on all factors. This revealed a significant main effect of Colour, *F*(1, 29) = 13.4, *p* = .001, $\eta_{p}^{2}$ = .316, indicating faster responses when the colour repeated compared with when the colour changed (378 ms vs. 393 ms). The main effect of Duration was also significant, *F*(1, 29) = 16.8, *p* < .001, $\eta_{p}^{2}$ = .367, indicating faster responses when the duration repeated compared with when the duration changed (383 ms vs. 388 ms). The factor Location was not significant (*F* = 2.35, *p* = .136, $\eta_{p}^{2}$ = .075).

**Table 2.** Mean response times in ms, as a function of Colour, Location, and Duration for Experiment 4.

	Colour repetition		Colour shift	
	Location repetition	Location shift	Location repetition	Location shift
RT				
Duration repetition	360 (10.7)	386 (9.10)	410 (10.5)	374 (8.21)
Duration shift	371 (10.4)	394 (10.2)	407 (9.91)	379 (9.82)

The respective standard errors are in brackets. RT: reaction time.

In line with Experiment 2, the Colour × Duration interaction reached significance, *F*(1, 29) = 8.39, *p* = .007, $\eta_{p}^{2}$ = .224, indicating partial repetition costs: Responses were faster when colour and duration were repeated compared with when colour repeated and duration changed (373 ms vs. 383 ms). The Bayes factor (BF_10_ = 8.27) of this interaction suggests substantial evidence for H_1_. Also in line with Experiment 3, the Location × Duration interaction was not significant (*F* = 0.201, *p* = .658, $\eta_{p}^{2}$ = .007). The Bayes factor (BF_01_ = 4.94) of this interaction suggests substantial evidence for H_0_. Furthermore, results showed a significant Colour × Location interaction, *F*(1, 29) = 113, *p* < .001, $\eta_{p}^{2}$ = .796, indicating faster responses for colour changes combined with a location change compared with a colour change combined with a location repetition (377 ms vs. 408 ms). The same applies to the full repetition of colour and location compared with the partial repetition of colour and location (366 ms vs. 390 ms). The three-way interaction Colour × Location × Duration was not significant (*F* = 2.41, *p* = .132, $\eta_{p}^{2}$ = .077).^7^

An analogous ANOVA for errors yielded a significant interaction Colour × Location, *F*(1, 29) = 48.2, *p* < .001, $\eta_{p}^{2}$ = .624, indicating partial repetition costs: The combination of a colour repetition and a location change was more error prone than a full repetition of colour and location (7.29% vs. 1.87%). The combination of a colour change and a location repetition was also more error prone than a complete alternation of colour and location (8.58% vs. 2.76%). None of the other effects was significant (all *F*s < 2.66 and *p*s > .114). As in the experiments before, the mean error rate (5.11%) was low.

The results of Experiment 4 brought up partial repetition costs between colour and duration but not between location and duration, thereby confirming results from Experiments 2 and 3. This strengthens our assumption that a context of location changes modulates the binding effects between colour and duration and may also explain the absence of an effect in Experiment 1 where no location changes were involved.

## General discussion

This study aimed to determine whether temporal stimulus features are integrated into visual event files. The results suggest that the underlying mechanisms for integrating temporal stimulus features in the visual context are far more complex than for integrating temporal stimulus features in the auditory context. The results of all four experiments show a pattern that task-irrelevant duration can be integrated as a stimulus feature in visual event files depending on two criteria: The experimental context holds the possibility of a change of location of the target stimulus (as it was the case in Experiments 2 and 4) and location itself is not response relevant (as it was in Experiment 3).

In Experiment 1, the colour stimulus was always presented at the same location, resulting in no interaction between colour and duration, indicating no binding of colour and duration. Experiment 2 embedded the colour stimuli in a potentially relevant context (traffic light), with the expectation that duration and colour would be bound if the duration of the visual stimulus suggests an informative character (traffic light) but still is not task-relevant. Contrary to our expectation, duration was bound to colour independently of the framing condition. Experiment 3 was designed to clarify whether duration might be bound to location because in Experiment 2 each colour change was associated with a location change, making colour and location not clearly separable. The results showed no binding of duration to location and thus no integration of duration into visual event files. It can thus be assumed that there was indeed a binding of duration to colour and not to location in Experiment 2. In Experiment 4, the experimental setup allowed the independent manipulation of colour and location and the results revealed partial repetition costs between colour and duration confirming the results of Experiment 2. These costs did not show up between location and duration, confirming the results of Experiment 3. This furthers the assumption that although duration is not bound to location itself (Experiment 3), a context of location changes modulates the binding effects between colour and duration and could also explain the absence of binding effects in Experiment 1.

It is well established that perceptual grouping leads to stronger binding effects (e.g., Frings & Rothermund, 2011; Laub et al., 2018; Moeller et al., 2012; Schmalbrock et al., 2022). One could argue that the results of Experiment 2 are the consequence of grouping stimulus features by the traffic light context. The absence of the three-way interaction contradicts this assumption. Partial repetition costs and thus binding were found in both the traffic light task and the circle task. As the circle task did not include any grouping element, we suppose that the partial repetition costs in Experiment 2 cannot be entirely explained by perceptual grouping.

A common question that arises when there are no partial repetition costs is whether it is due to a lack of binding of the features in trial *n* − 1 or a lack of retrieval of the features in trial *n* (e.g., Frings et al., 2020). More specifically, the question is whether there was no binding/integration of the features colour and duration (or Experiment 3 location and duration) or whether a repetition of one or both features did not result in a retrieval of the previous event file. Recent findings by Kikumoto and Mayr (2020) seem to speak against this latter assumption. In their study, the authors were able to demonstrate the existence of event files at the neural level, using a two-stage representational similarity analysis. Their results showed temporally precise evidence that bindings are already formed during action selection in trial *n* − 1. Direct comparison of bindings from trial to trial showed that the strength of the binding was a robust predictor of upcoming response behaviour, and the stronger the binding in trial *n* − 1, the greater the partial repetition costs in trial *n*. That is, results could be interpreted in the sense that if the binding is strong enough, it will also be retrieved. Applied to our results presented here, this would mean that there was no integration of colour and duration (or Experiment 3 location and duration) into an event file in the design and setting used. At the very least, the binding of the features in trial *n* − 1 was probably too weak to exert its influence on the response behaviour in the subsequent trial *n*.

Consistent with our differential findings regarding the role of location for the integration of duration in event files, the role of location in binding processes is also discussed controversial in the literature. Location plays a major role in binding theories. Most notably, location-based theories and object-indexing theories proclaim that location has an essential and functional role in binding visual features. One must first locate the object to focus attention on it to then integrate the associated features (Hommel, 2004; Kahneman et al., 1992; Kovacs & Harris, 2019; Leslie et al., 1998; Treisman, 1988; Treisman & Gelade, 1980). However, there are also opinions that claim location does not play a major role in binding processes (e.g., Allen et al., 2015). Nevertheless, the existing binding effects in Experiments 2 and 4 are more consistent with location-based theories, which proclaim that an object must first be located in order for its associated features to be integrated. The fact that the stimuli in Experiments 2 and 4 could appear either above or below the fixation cross might have elicited the process of “localising” and thus favoured the subsequent integration of the stimulus features colour and duration. Following this train of thought, the process of “localising” would not have been necessary in Experiment 1 because the stimuli were presented steadily in the centre and thus the subsequent process of integrating colour and duration did not occur. Contrary to this consideration, however, are the results of Zehetleitner et al. (2012), who presented their participants a centrally appearing stimulus (Experiment 1b: striped blue square or striped green circle) to which orientation was to be responded to. For this combination of purely static features, with no need for primary localisation of the stimulus, a pattern of partial repetition costs emerged. All this suggests that location does play a role for the integration of stimulus duration into visual event files, but in a new as yet unexplained way.

The results of Experiment 3, in which participants were asked to respond to the stimulus location, showed no binding of duration and location. Based on the results of a series of experiments, Hommel (2007) concluded that the binding of location as a stimulus feature (S) to response (R) only occurs when the location is task-relevant or the response set is spatially defined (left–right). He used a prime-probe paradigm in which participants were asked to make a pre-cued response (R1) to the first stimulus (S1) and to make a free-choice response (R2) to the second stimulus (S2). Feature integration was measured by response repetition rate. Results showed a higher response repetition rate, in the case of stimulus location repetition, when the location was task-relevant or the response set was spatially defined (as compared with nonspatially defined). Because in this study in Experiment 3 the location was task-relevant (and the response set spatially defined), the lack of interaction between location and duration cannot be explained by absent task-relevance of location. It rather indicates that duration is actually not bound to location.

Furthermore, it would be reasonable to consider whether the missing partial repetition costs in Experiments 1 and 3 can be explained by the task irrelevance of the duration feature. Findings regarding the integration of task-irrelevant features are inconsistent. There are studies showing comparable binding effects for both task-relevant and task-irrelevant features (e.g., Hommel & Colzato, 2004; Moeller et al., 2012; Moeller, Frings, & Pfister, 2016) with comparable strength of binding (e.g., Moeller, Frings, & Pfister, 2016) and there are findings showing weaker binding of irrelevant features and only binding of certain irrelevant features (e.g., Hommel, 1998; Zmigrod & Hommel, 2009). Singh and Frings (2018) found that several irrelevant features are reliably bound when there is little to no visual noise and thus no “competition” of irrelevant features. Once visual noise increases, “competition for binding” also increases and results in reduced binding effects. In the present study, the feature of interest, namely, duration, was always irrelevant. In Experiments 1 and 3 (no partial repetition costs), there was only one relevant feature (Experiment 1: colour, Experiment 3: location) and one irrelevant feature (duration) in each case. The visual noise should therefore have been rather low and the feature duration had no other “competitor” in the battle for binding. Nevertheless, there were no partial repetition costs and thus no evidence of binding of the features. In Experiments 2 and 4 (partial repetition costs), there were, in addition to the one relevant feature colour, two irrelevant features each, duration and location. Although this increased the visual noise, we revealed partial repetition costs. Thus, visual noise does not provide a sufficient explanation for the lack of partial repetition costs in Experiments 1 and 3, even though duration was always irrelevant in the present study. Importantly, Bogon, Thomaschke, and Dreisbach (2017) already demonstrated that task-irrelevant duration was integrated into auditory event files. Nevertheless, considering the differences of the visual and auditory domains, we assume that the integration of stimulus duration into visual event files might be easier to demonstrate when stimulus duration is relevant.

It should be noted that due to the design used in the present study, a repetition or change of the task-relevant feature (colour or location) was also a repetition or change of the response (left–right) at the same time. Because of this constellation, it cannot be said with certainty whether duration was bound (or not bound) to the other stimulus feature or to the response. This design-specific question would have to be investigated in subsequent research using, for example, a many-to-one mapping design (e.g., Giesen & Rothermund, 2011; Moeller & Frings, 2014; Moeller, Pfister, et al., 2016) in which independent modulation of stimulus and response feature is possible (at least to some degree). Nevertheless, the essential question of this study was whether duration as a temporal stimulus feature can be integrated into visual event files. As the response as a feature also belongs to an event file (see Hommel, 1998, 2004), answering the question was possible using the present design.

Another consideration for the question why partial repetition costs did not occur in Experiment 1 but did in Experiment 4 may be different underlying organisational structures of binding. Singh and Frings (2020) found that the binding structure is more object-based in the case of location variation and more feature-based in the case of constant location. Moreover, stimulus and location repetitions led to larger binding effects, especially when the stimulus location was variable. These findings fit with the results of the present study: In Experiment 1, the stimulus location never changed; consequently, the underlying binding structure could be feature-based. If the location of a stimulus is always predictable, it loses relevance/need for feature integration. Although this does not necessarily imply that feature binding can never occur with constant stimulus location, in the case of Experiment 1 of the present study, it could have resulted in no binding between the relevant feature colour and the irrelevant feature duration. The binding might have been too weak to show in terms of partial repetition costs. In Experiment 4, the location varied and partial repetition costs were found, indicating a binding of colour and duration. Furthermore, it was found that the significant Colour × Duration interaction only occurred with location repetition (note: the three-way interaction between Colour × Location × Duration was not significant, so this particular result should be considered with caution), which in turn fits with Singh and Frings’s (2020) findings that stimulus and location repetition lead to stronger binding effects. This could possibly also explain the occurring binding of colour and duration in Experiment 2, since here, due to the construction of a traffic light, the colour stimuli could appear above or below (variation of the location). However, because in Experiment 2 colour and location cannot be considered separately (yellow always above, green always below), this assumption cannot be confirmed with certainty. Consequently, for the integration of duration as an irrelevant stimulus feature in the visual context, the presence of location variation seems necessary (results Experiments 1, 2, & 4), but only if they are task-irrelevant (results Experiment 3).

So far, the results of this series of experiments suggest that the integration of duration into visual event files critically depends on the context of a location change. But what makes the location change of a stimulus more prone to binding temporal features? A possible reason might be motion perception: Whenever a stimulus changes its location, this induces some kind of motion perception (Albright & Stoner, 1995; Johansson, 1975). And motion perception itself is highly dependent on time characteristics as they are indicative of speed. Pictures on the wall usually do not move, but a picture on a passing van does move and the movement itself carries important information about speed (and the possibility to cross the road without risk). That is, duration acquires a functional role through motion (change of location) and might thereby also increase the perceived informational value of the entire stimulus (including the duration feature). Therefore, it might in fact be functional that duration is integrated into moving visual events but not into static visual features.

This need for additional context for the integration of temporal stimulus features in the visual modality is not necessary for the integration of temporal stimulus features in the auditory modality (see Bogon, Thomaschke, & Dreisbach, 2017). The design used in Experiment 1 of this study was equivalent to the design applied by Bogon, Thomaschke, and Dreisbach (2017). They used a sequential two-factorial design in both experiments, through which they were able to demonstrate the binding of pitch and duration (Experiment 1) and loudness and duration (Experiment 2). Based on the fact that this study could not demonstrate the binding of colour and duration in Experiment 1, despite using the equivalent design, these findings suggest that the integration of temporal stimulus features into event files differs between auditory and visual modalities. Such modality differences with respect to binding effects have been shown before (Mayr & Buchner, 2010; Schöpper & Frings, 2022). In addition, there are modality differences in time perception (see Wearden & Jones, 2021, for a review), such as auditory events being subjectively estimated to be longer than visual events, although both are objectively of the same duration (Goldstone & Lhamon, 1974; Walker & Scott, 1981; Wearden et al., 1998). The results of this study fit into this pattern of modality differences. The integration of temporal stimuli into event files has been found to be more complex in the visual modality than in the auditory modality. Moreover, the binding effects of temporal stimuli appear to be weaker in the visual context (this study, Experiments 2 & 4; average: $\eta_{p}^{2}$ = .22) than in the auditory context (Bogon, Thomaschke, & Dreisbach, 2017; average $\eta_{p}^{2}$ = .47). Further research could address specific differences in the integration of temporal stimulus features in event files across modalities.

In conclusion, research on the integration of temporal features is on the rise (temporal stimulus feature: Bogon, Thomaschke, & Dreisbach, 2017; temporal response feature: Mocke et al., 2022; Pfister et al., 2022; temporal expectancy: Schmalbrock & Frings, 2022) and brings valuable new insights that can also lead to further explanatory approaches across topics (e.g., temporal Simon effect by Kunde, 2003; Kunde & Stöcker, 2002). In this study, we were able to show that task-irrelevant duration as a stimulus feature, despite its underlying dynamic structure, can be integrated into visual event files. The occurrence of these stimulus-stimulus binding effects seems to be related to a context of location changes. Nevertheless, the findings still leave open questions regarding the exact mechanisms of feature integration of temporal stimulus features in a visual context. Further research is needed to explore the possibilities and limitations of this feature integration.

## Notes

1 Due to the technical equipment and the rounding algorithms of E-Prime, the actual presentation durations were as follows: for “short,” *M* = 33.38 ms, *SE* = 0.008 ms, *min* = 33 ms, *max* = 34 ms, and for “long,” *M* = 300.4 ms, *SE* = 0.009 ms, *min* = 300 ms, *max* = 301 ms.

2 The categorization of Bayes factors is based on the work of Wagenmakers et al. (2011) (BF_01_) and Wetzels et al. (2011) (BF_10_). Wagenmakers et al.’s categorization for the BF_01_ is as follows: >100 “Extreme evidence for H_0_”; 30–100 “Very strong evidence for H_0_”; 10–30 “Strong evidence for H_0_”; 3–10 “Substantial evidence for H_0_”; 1–3 “Anecdotal evidence for H_0_”; 1 “No evidence”; 0.33–1 “Anecdotal evidence for H_1_”; 0.1–0.33 “Substantial evidence for H_1_”; 0.033–0.1 “Strong evidence for H_1_”; 0.01–0.033 “Very strong evidence for H_1_”; <0.01 “Extreme evidence for H_1_.” Wetzel et al. categorise the BF_10_ accordingly in reverse and uses the term “Decisive” for the “Extreme” categories (from Wagenmakers et al.).

3 Due to the technical equipment and the rounding algorithms of E-Prime, the actual presentation durations were as follows: for “short,” *M* = 23.89 ms, *SE* = 0.011 ms, *min* = 23 ms, *max* = 27 ms, and for “long,” *M* = 306.3 ms, *SE* = 0.008 ms, *min* = 306 ms, *max* = 318 ms.

4 To make sure that the significant Colour × Duration interaction could not be ascribed to the increased number of trials in Experiment 2 (6 × 80 trials) compared with Experiment 1 (3 × 80 trials), we looked into this interaction separately for the standard and the traffic light conditions. The Colour × Duration interaction was significant for both conditions: standard: *F* (1, 35) = 4.95, *p* = .033, $\eta_{p}^{2}$ = .124, BF_10_ = 1.8, and traffic light: *F*(1, 35) = 10.4, *p* = .003, $\eta_{p}^{2}$ = .229, BF_10_ = 17.9.

5 Due to the technical equipment and the rounding algorithms of E-Prime, the actual presentation durations were as follows: for “short,” *M* = 23.32 ms, *SE* = 0.007 ms, *min* = 23 ms, *max* = 24 ms, and for “long,” *M* = 305.8 ms, *SE* = 0.009 ms, *min* = 305 ms, *max* = 306 ms.

6 Due to the technical equipment and the rounding algorithms of E-Prime, the actual presentation durations were as follows: for “short,” *M* = 23.31 ms, *SE* = 0.005 ms, *min* = 23 ms, *max* = 24 ms, and for “long,” *M* = 305.8 ms, *SE* = 0.005 ms, *min* = 305 ms, *max* = 306 ms.

7 Equivalent to Experiment 2, we looked at the Colour × Duration interaction separately for location repetitions and location changes to prevent the Colour × Duration interaction from occurring only because of the increased trial number (6 × 80 trials) compared with Experiment 1 (3 × 80 trials). The Colour × Duration interaction was significant for the location repetition condition, *F*(1, 29) = 6.09, *p* = .020, $\eta_{p}^{2}$ = .174, BF_10_ = 3.18, and not significant for the location shift condition, *F* = 0.586, *p* = .450, $\eta_{p}^{2}$ = .020, BF_01_ = 4.1.
